# ELIXIR and Toxicology: a community in development

**DOI:** 10.12688/f1000research.74502.1

**Published:** 2021-11-08

**Authors:** Marvin Martens, Rob Stierum, Emma L. Schymanski, Chris T. Evelo, Reza Aalizadeh, Hristo Aladjov, Kasia Arturi, Karine Audouze, Pavel Babica, Karel Berka, Jos Bessems, Ludek Blaha, Evan E. Bolton, Montserrat Cases, Dimitrios Ε. Damalas, Kirtan Dave, Marco Dilger, Thomas Exner, Daan P. Geerke, Roland Grafström, Alasdair Gray, John M. Hancock, Henner Hollert, Nina Jeliazkova, Danyel Jennen, Fabien Jourdan, Pascal Kahlem, Jana Klanova, Jos Kleinjans, Todor Kondic, Boï Kone, Iseult Lynch, Uko Maran, Sergio Martinez Cuesta, Hervé Ménager, Steffen Neumann, Penny Nymark, Herbert Oberacher, Noelia Ramirez, Sylvie Remy, Philippe Rocca-Serra, Reza M. Salek, Brett Sallach, Susanna-Assunta Sansone, Ferran Sanz, Haralambos Sarimveis, Sirarat Sarntivijai, Tobias Schulze, Jaroslav Slobodnik, Ola Spjuth, Jonathan Tedds, Nikolaos Thomaidis, Ralf J.M. Weber, Gerard J.P. van Westen, Craig E. Wheelock, Antony J. Williams, Hilda Witters, Barbara Zdrazil, Anže Županič, Egon L. Willighagen

**Affiliations:** 1 Department of Bioinformatics - BiGCaT, Maastricht University, Maastricht, 6229 ER, The Netherlands; 2 Risk Analysis for Products In Development (RAPID), Netherlands Organisation for applied scientific research TNO, Utrecht, 3584 CB, The Netherlands; 3 Luxembourg Centre for Systems Biomedicine (LCSB), University of Luxembourg, Belvaux, 4367, Luxembourg; 4 Maastricht Centre for Systems Biology (MaCSBio), Maastricht University, Maastricht, 6229 EN, The Netherlands; 5 Laboratory of Analytical Chemistry, Department of Chemistry, National and Kapodistrian University of Athens, Athens, 15771, Greece; 6 Institute of Biophysics and Biomedical Engineering, Bulgarian Academy of Sciences, Sofia, 1113, Bulgaria; 7 Department Environmental Chemistry, Swiss Federal Institute of Aquatic Science and Technology, Dübendorf, 8600, Switzerland; 8 Université de Paris, Paris, F-75006, France; 9 RECETOX, Faculty of Science, Masaryk University, Brno, Czech Republic; 10 Department of Physical Chemistry, Palacky University Olomouc, Olomouc, 77146, Czech Republic; 11 Health Unit, Health, Mol, 2400, Belgium; 12 National Center for Biotechnology Information, National Library of Medicine, National Institutes of Health, Bethesda, MD 20894, USA; 13 Chemotargets SL, Barcelona, 08028, Spain; 14 School of Science, GSFC University, Gujarat, 391750, India; 15 Forschungs- und Beratungsinstitut Gefahrstoffe (FoBiG) GmbH, Freiburg im Breisgau, 79106, Germany; 16 Seven Past Nine, Cerknica, 1380, Slovenia; 17 AIMMS Division of Molecular Toxicology, Vrije Universiteit, Amsterdam, 1081 HZ, The Netherlands; 18 Department of Toxicology, Misvik Biology, Turku, 20520, Finland; 19 Institute of Environmental Medicine, Karolinska Institute, Stockholm, 17177, Sweden; 20 Department of Computer Science, Heriot-Watt University, Edinburgh, UK; 21 ELIXIR Hub, Wellcome Genome Campus, Cambridge, CB10 1SD, UK; 22 Department Evolutionary Ecology & Environmental Toxicology (E3T), Goethe-University, Frankfurt, D-60438, Germany; 23 Ideaconsult Ltd., Sofia, Bulgaria; 24 Department of Toxicogenomics, Maastricht University, Maastricht, 6200 MD, The Netherlands; 25 MetaboHUB, French metabolomics infrastructure in Metabolomics and Fluxomics, Toulouse, France; 26 Toxalim (Research Centre in Food Toxicology), Université de Toulouse, Toulouse, France; 27 Scientific Network Management SL, Barcelona, 08015, Spain; 28 Faculty of Pharmacy, Malaria Research and Training Center, Bamako, BP:1805, Mali; 29 School of Geography, Earth and Environmental Sciences, University of Birmingham, UK, Birmingham, B15 2TT, UK; 30 Department of Chemistry, University of Tartu, Tartu, 50411, Estonia; 31 Data Sciences and Quantitative Biology, Discovery Sciences, AstraZeneca, Cambridge, UK; 32 Institut Français de Bioinformatique, Evry, F-91000, France; 33 Bioinformatics and Biostatistics Hub, Institut Pasteur, Paris, F-75015, France; 34 Research group Bioinformatics and Scientific Data, Leibniz Institute of Plant Biochemistry, Halle, 06120, Germany; 35 Institute of Legal Medicine and Core Facility Metabolomics, Medical University of Innsbruck, Innsbruck, A-6020, Austria; 36 Institut d'Investigacio Sanitaria Pere Virgili-Universitat Rovira i Virgili, Tarragona, 43007, Spain; 37 Data Readiness Group, Department of Engineering Science, University of Oxford, Oxford, UK; 38 International Agency for Research on Cancer, World Health Organisation, Lyon, 69372, France; 39 Department of Environment and Geography, University of York, UK, York, YO10 5NG, UK; 40 Research Programme on Biomedical Informatics (GRIB), Hospital del Mar Medical Research Institute (IMIM), Department of Experimental and Health Sciences, Pompeu Fabra University, Barcelona, 08003, Spain; 41 National Technical University of Athens, Athens, 15780, Greece; 42 Helmholtz Centre for Environmental Research - UFZ, Leipzig, 04318, Germany; 43 Environmental Institute, Koš, 97241, Slovakia; 44 Department of Pharmaceutical Biosciences and Science for Life Laboratory, Uppsala University, Uppsala, SE-75124, Sweden; 45 School of Biosciences, University of Birmingham, UK, Birmingham, B15 2TT, UK; 46 Division of Drug Discovery and Safety, Leiden Academic Center for Drug Research, Leiden, 2333 CC, The Netherlands; 47 Department of Respiratory Medicine and Allergy, Karolinska University Hospital, Stockholm SE-141-86, Sweden; 48 Department of Medical Biochemistry and Biophysics, Karolinska Institute, Stockholm, 17177, Sweden; 49 Center for Computational Toxicology and Exposure, United States Environmental Protection Agency, Research Triangle Park, NC 27711, USA; 50 Department of Pharmaceutical Sciences, University of Vienna, Vienna, 1090, Austria; 51 Department Biotechnology and Systems Biology, National Institute of Biology, Ljubljana, 1000, Slovenia

**Keywords:** Toxicology, ELIXIR, interoperability, FAIR

## Abstract

Toxicology has been an active research field for many decades, with academic, industrial and government involvement. Modern omics and computational approaches are changing the field, from merely disease-specific observational models into target-specific predictive models. Traditionally, toxicology has strong links with other fields such as biology, chemistry, pharmacology and medicine. With the rise of synthetic and new engineered materials, alongside ongoing prioritisation needs in chemical risk assessment for existing chemicals, early predictive evaluations are becoming of utmost importance to both scientific and regulatory purposes. ELIXIR is an intergovernmental organisation that brings together life science resources from across Europe. To coordinate the linkage of various life science efforts around modern predictive toxicology, the establishment of a new ELIXIR Community is seen as instrumental. In the past few years, joint efforts, building on incidental overlap, have been piloted in the context of ELIXIR. For example, the EU-ToxRisk, diXa, HeCaToS, transQST, and the nanotoxicology community have worked with the ELIXIR TeSS, Bioschemas, and Compute Platforms and activities. In 2018, a core group of interested parties wrote a proposal, outlining a sketch of what this new ELIXIR Toxicology Community would look like. A recent workshop (held September 30th to October 1st, 2020) extended this into an ELIXIR Toxicology roadmap and a shortlist of limited investment-high gain collaborations to give body to this new community. This Whitepaper outlines the results of these efforts and defines our vision of the ELIXIR Toxicology Community and how it complements other ELIXIR activities.

## Introduction of the ELIXIR Toxicology Community

Toxicology as a field tries to understand the negative consequences that may arise from the interactions of chemicals with living organisms. In this ELIXIR (European life-sciences Infrastructure for biological Information) ^1^ Community, the focus will be primarily the protection of human health. There are a number of both chemical and biological “interoperability” issues key to the toxicology field that translate into data interoperability issues. These include the connection between the action and activity of a particular chemical compound to its effective amount available at a biological target (the link between toxicodynamics and toxicokinetics). Typically this is also a link between biological data analysis (including large-scale multi-omics) and kinetic modelling. Other examples include interactions between a compound and its target (a protein, nucleic sequence or membrane structure for instance). This is primarily based on the interplay between chemistry (the chemical structure and, for example, its related properties in terms of functional groups, charge, shape and related binding affinity) and biochemistry (like biomolecular 3D structures). Also, mixture toxicity needs to be considered as combinations of chemicals with synergistic or antagonistic behaviour, or a combination thereof. While chemicals with similar modes of action may act in terms of concentration addition, those with different modes may rather act according to independent action. ^2^^,^^3^ Substances with low toxicity may interact in concentration addition rather than as excess toxicity drivers of one compound. Often there is a need to translate the knowledge about one compound into knowledge about other compounds, where approaches like quantitative structure-activity relationships (QSARs) help to elucidate this knowledge and predict required property and toxicity, and in more general when “read-across approaches” come into play that are based on chemical data only, biological data only, or are hybrid. ^4^^,^^5^ These again need detailed information about the relationships between related chemical compounds, specific properties and toxicological endpoints and, when considering chemical-biological interactions, details regarding both the chemical structures and adequate descriptions of the biological targets. Since toxicological endpoints can be represented in a myriad of ways, the toxicological effect data are often scattered over multiple repositories and databases hosting different types of data; i.e. chemical structures, toxicity data ( *in vivo* and *in vitro*), biological target details, and omics data. This itself is not a problem, but the separation and segregation make the data difficult to find and to connect. Currently, many of these deposition databases (where datasets can be archived) do not provide adequate descriptions regarding typical toxicological study designs and parameters, quality control, data acceptance criteria, or even clear identification of the compounds tested. This all adds to the need for an adequate FAIRification process, to make toxicology data more Findable, Accessible, Interoperable and Reusable (FAIR - see Go-FAIR). ^6^^,^^7^

The field of toxicology would certainly benefit from clear, standardized guidelines for data capture, approaches to integrate and connect across multiple databases, clear data licensing for these data repositories, and tools that support accessing the data (as being developed by the ELIXIR Converge data brokerage work). Existing study capture tools can be extended with templates defined for toxicology, which end up in a central place ( *e.g.* Biosamples ^8^) with links to omics and other data distributed over technology-specific deposition databases and BioStudies. ^9^ Relevant portals ( *e.g.* FAIRsharing.org ^10^^,^^11^) should then be able to identify these studies, also linking to existing but scattered toxicology databases.

Risk assessment, consisting of hazard identification, characterisation and risk evaluation in relation to exposure, involves expert opinions based on discussions and data interpretation. Streamlining this process is important also because of the huge number of chemicals known and produced in chemistry, biotechnology or food production, with over 350,000 chemicals documented on the global market. ^12^ However, expert evaluation is and will remain crucial and calls for extensive data provenance both for the actual data (how was it measured, where was it published, whom to give credit for it), and for the risk assessments themselves. Interestingly this process stumbles on problems and solutions that have much in common with other fields, where part of the data need to be hidden and other parts can be publicly accessed, as observed in pilot approaches in genetics, or patient data repositories.

Toxicology, while established as discipline, is also rapidly developing in areas, where, for example, new molecular methods to describe the adverse outcome of exposure to a toxic substance are not yet fully established. This offers opportunities for integration in systems biology approaches building on molecular pathway descriptions that benefit from modern network biology approaches. Compound profiling, where, for example, using omics methods to characterize the effect of compounds on cells, are generally useful for categorizing compounds, or for the development of predictive pathway models. For such studies, the availability of high-quality annotations for compounds is of paramount importance to enable the use of omics profiles in toxicology.

Toxicology is also an applied field. There are important applications of toxicity tests in the regulatory field and large amounts of data are collected for that purpose, often for different (governmental) agencies. Making this data more Findable and Reusable is often seen as an important way to reduce both animal testing and the cost of registration of new compounds. If some data is not available to the public due to ownership by companies or other constraints, indexes should be developed to enable the use of this data in an aggregated form (such as the SPIN index of the Swedish Chemicals agency ( KEMI) and other Nordic chemical agencies). Typically, regulatory use requires very precise and rigid descriptions of protocols, including reporting methods. On the other hand, better insights into the toxicological mechanisms, including interactions between chemicals, benefit from more innovative research methods ( *e.g.* single-cell omics, induced stem cell applications, imaging methods) and from creative development of new analysis methods. While it is beneficial to combine results from both types of approaches (termed New Approach Methods (NAM) in the regulatory field), the corresponding interoperability issues are often quite different.

## User community

Europe is steadily increasing demands on the risk assessment of chemicals, drugs, cosmetics ingredients and nanomaterials to lead to safer products, resulting in a strong toxicology research community with sub-communities in, for example, the drug development, environmental, nanomaterial and rare-disease areas. Recent changes in European law for animal testing and new demands for testing of lower-volume chemicals and nanomaterials have triggered large-scale research into alternative testing approaches. These activities not only produce new biological and mechanistic insights, but also large amounts of new data, which have to be managed and shared for re-usage to avoid unnecessary duplication of experiments and, in this way, reduce animal testing. The goal is that the combination of data from integrated *in vitro* and *in silico* approaches will support (ultimately personalized) risk/benefit health analysis, safer drug innovation with fewer needs to withdraw after registration, a fact-based perception of chemical safety, safe-by-design nanomaterials and sustainable and safe economies.

The European Union supports toxicological and risk assessment projects with various funding programs. Recently, large collections of data have been released, resulting from research clusters, such as SEURAT-1, ^13^ the EU NanoSafety Cluster (NSC) with NANoREG, EU-ToxRisk, ^14^ the NORMAN Network, and the EU Innovative Medicines Initiative (IMI) funded projects related to drug toxicology, including eTOX, ^15^ eTRANSAFE, ^16^ and Eurion. ^17^ Data from these and other projects are becoming available, sometimes as Open Data ( *e.g.* NANoREG) and sometimes as FAIR data. An example of the latter is European REACH data, which has recently been made FAIR by the Cefic-LRI-funded project AMBIT-LRI. ^18^ Furthermore, the FAIRplus project is collaborating with eTOX on making their data more FAIR, and the new Precision Toxicology will develop a data commons following the FAIR principles.

However, there are a few opportunities for data handling that need to be taken. ^19^^,^^20^ Recent studies show how powerful the combination of toxicology information and omics data is, ^21^^,^^22^ but to be able to obtain the statistical significance to draw these conclusions, data from the US and Japan had to be combined. In contrast to large data sets like DrugMatrix, ^23^ ToxCast/Tox21, ^24^ and TG-GATEs ^25^ from these countries, data from European projects is often not sufficiently integrated. Luckily, there are signs that the community is going in the right direction, *e.g.* the aforementioned data integration by diXa, ^26^ NANoREG and REACH. ^27^ With respect to the European Chemical Industry, various authors have been involved in other Cefic-LRI activities related to data management ( AIMT-3, AIMT-4).

In addition, the eTOX project ^28^ has established data integration approaches *e.g.* to enable the development of QSARs relating chemical structures to *in vivo* toxicopathological outcomes. As such, the project also delivered databases and approaches to ontology development, text mining approaches, and approaches for prediction of drug metabolism and pharmacokinetic features. Moreover, in 2017 the Organisation for Economic Co-operation and Development ( OECD) performed an online survey underscoring the fact that data integration for safety is of global concern for ultimate risk assessment. The purpose of the resulting knowledge base is the integration of eCHEMportal (The Global Portal to Information on Chemical Substances ^29^), IUCLID (International Uniform ChemicaL Information Database ^30^), and OECD’s QSAR Toolbox ^31^ supporting the development of Adverse Outcome Pathways and associated infrastructures ( AOP-Wiki). ^32^ Despite these positive developments, the ‘data integration struggle’ from various perspectives (omics, computational chemistry and more ‘conventional’ toxicological data within REACH and pharmaceutical industry setting) remains a challenge.

## Roadmap

The above initiatives are mainly driven by user communities themselves: chemical industry, funding agencies, pharmaceutical companies, governmental agencies and organisations such as Member State organisations, the OECD and non-governmental organisations (NGOs). ELIXIR can contribute strongly to the existing infrastructure projects from a cheminformatics and bioinformatics perspective, providing tools and guidelines, linking and harmonizing the ongoing activities, serving the toxicology users.

For future risk assessment paradigms solely based on human-derived models, and in this way of higher relevance for human adverse effects, ^33^ various data types will need to be integrated into and cross the conventional boundaries of risk assessment. This involves external exposure assessment ( *e.g.* via workplace or environmental modelling and measurements ^34^^,^^35^), internal exposure characterisation (ADME-TK (absorption, distribution, metabolism, and excretion - toxicokinetics) *e.g.* via modelling and biomarker-based detection), toxicodynamics on a molecular level, and cell and systems biology. In this way, more data-driven mechanism-based evaluations and supporting data can integrate into regulatory risk assessment. There will not only be more but also more diverse data as *e.g.* internal exposure data may be inferred from biomonitoring data and/or physiologically-based toxicokinetic modelling to estimate target dose available at the active sites involved in the molecular initiating events of Adverse Outcome Pathways (AOPs).

Another relevant topic is the concept of the exposome, ^36^^–^^38^ which aims at characterising lifetime exposure (not only to chemicals in the narrowest sense, but also dietary components, lifestyle factors, environmental exposures, etc) in relation to health outcome. Often, vulnerable periods of life (infancy, childhood, and old age) are investigated, and the evaluation also integrates epidemiology. This is one clear demonstration of the trend that the previously distinct areas of toxicology, drug and product design and personalized/precision medicine ^39^ but also environment and health and epidemiology are moving closer together. Data sharing will be increasingly necessary across these disciplines. High throughput data analysis in exposomics, for instance, shares many parallels with metabolomics and other higher level omics analyses, with an added layer of chemical complexity on top. ^38^

The toxicology community is large and well established. The current list of proponents of an ELIXIR Toxicology Community only reflects a subset of a much larger community with a lot of commitments to, and activities around open collaboration. It has clear omics, knowledge management and data infrastructure needs to accommodate the increasing wish to predict toxicology without animal testing ( *e.g.* in SEURAT-1, EU-ToxRisk, eTOX OBERON ^40^). Foreseeing this need for better infrastructures, the community has previously contacted ELIXIR for collaboration. Various domain-specific projects exist that service the toxicology community with computational and database knowledge ( OpenRiskNet, NanoCommons) that can translate ELIXIR knowledge to the respective communities. These infrastructure projects are the successors of research projects focusing on data management, including diXa, ^26^ ToxBank ^41^ and eNanoMapper. ^42^

To benefit the research community, small and medium-sized enterprises (SMEs) and larger industry, and to enable further future support to regulatory applications and upcoming calls ( *e.g.* Green Deal ^43^^,^^44^), we need to reach an inclusive ecosystem of data, evaluation and modelling tools. The current separate consortia from different toxicity-related and neighbouring disciplines already work towards data and knowledge that is FAIR. ^6^ After all, these aspects are essential to efficiently assess the risk of new compounds and materials, as well as combined risks of current stressors ( *e.g.* under the exposome concept). To further accelerate these activities, more toxicology-related data and knowledge need to be linked, such as on physiologically based toxicokinetic (PBTK) modelling, biological pathways describing affected metabolism and cell biology processes, metabolism, metabolic models and metabolism predictions, drug-response, omics ( *biological identity*), chemical structures and associated metadata (use, hazard, transformations), QSARs, AOPs, REACH dossiers, etc. Simultaneously, an extension towards the human (preclinical toxicology) discipline should be initiated, in which exposure data are combined with internal exposure and early biomarkers of effect data ( *e.g.* from the European Human Biomonitoring Initiative ( HBM4EU) ^45^ and environmental data from NORMAN ^46^^,^^47^) towards pathways of toxicity. Interoperability with the standardization efforts of clinical research by CDISC is important.

However, to reach such interoperable toxicology, resources need to be better integrated. Despite the work of many projects, their FAIR features can still be improved and applying newly developed FAIR metrics will help steer this. ^48^ Even though there is overlap in content with existing ELIXIR Communities ( Table 1, key demands specifically fostering the integration for interoperable toxicology and risk assessment include the following roadmap ^19^^,^^20^):

• chemical structure interoperability challenges ( *e.g.* links to ELIXIR Metabolomics Community ^49^)
• metadata, open standards ( *e.g.* links to ELIXIR Interoperability Platform, and TeSS ^50^)
• continued ontology development ( *e.g.* links to ELIXIR Interoperability Platform and Ontology Lookup Service)
• interoperable computation ( *e.g.* links to the Galaxy ^51^ and bio.tools ^52^ communities)
• interactions with other ELIXIR Core Data Resources ( *e.g.* Ensembl and Europe PMC)
• interactions with other communities, including nanomedicine and health
• deployment of existing tools and modelling approaches on the ELIXIR Compute infrastructure (also allowing future growth towards risk estimations needing Monte Carlo approaches)
• integration of ELIXIR AAI
• InChI implementation for small molecule data
• Spectral database functionality (open implementations)

**Table 1.** Overlap of the proposed ELIXIR Toxicology Community with ELIXIR platforms and communities.

	Overlap
**Platforms**	
Tools	Semantic annotation of services ( *e.g.* bio.tools ^52^). Alignment with BioContainers ^62^ to make toxicology reproducible and redeployable ( *e.g.* OpenRiskNet)
Data	BioStudies was co-developed by the CHEMBL-EBI team, and builds on ArrayExpress, ^63^ an ELIXIR Core Data Resource. Better adoption of the core resources. Sharing toxicology workflows on WorkflowHub
Compute	ELIXIR Authentication and Authorisation Infrastructure (AAI) is used by OpenRiskNet. Better and more sustainable compute infrastructure reuse of core elements (ELIXIR AAI, modelling toolsets)
Interoperability	FAIR and Research Data Management (RDM) standards have already been adopted by various projects. Registries of toxicology tools need integration with FAIRsharing; There is a huge identifier mapping service need (also for ontology mapping and chemical (sub)structures), Common Workflow Language (CWL) ^69^ as interoperability for workflow, and standardized data exchange formats like the Investigation-Study-Assay (ISA) standard. ^64^
Training	Bioschemas annotated tutorials. Several projects have training tasks that could be added to TeSS, already partially automated.
**Communities**	
3D-bioinfo & Intrinsically Disordered Proteins (IDP)	Structural information about molecular actions of toxic compounds, molecular initiating events in AOPs
Galaxy	Application Programming Interface (API) standards and ontological annotation of APIs
Marine Metagenomics	Ecotoxicology data reflects effects on populations of species
Plant Sciences	Ecotoxicology data reflects effects of toxicants on plants
Metabolomics	Toxicants Chemical identifiers, data standards, data repository, common resources
Proteomics	Data standards and repository for proteomics data
Microbial Biotechnology	Chemical transformations in biological systems of toxicants
Federated Human Data & Human Copy Number Variation Community	Federated search, phenotype and genotype data
Rare Diseases	Shared biological pathways and interaction effects between rare diseases and exposure of toxicants

The concrete steps forward proposed in this roadmap include:

1. Leverage from open solutions (models, ontologies, educational material, standards etc) developed by past and ongoing toxicology and ELIXIR projects
2. Connect more closely with the core ELIXIR resources (FAIR data, database interoperability, etc), strengthen and connect the inclusive communities that have evolved over the past few years ( OpenTox, eNanoMapper, diXa, OpenRiskNet, NORMAN) and older communities like the Federation of European Toxicologists & European Societies of Toxicology ( EUROTOX), the Society of Environmental Toxicology and Chemistry ( SETAC) and European Environmental Mutagenesis and Genomics Society (EEMGS, formerly known as EEMS)
3. Develop open community standards to support common interest (ontologies, APIs, data formats, deposition databases and publication recommendations)

Specifically, we will continue to grow the list of involved toxicology research groups, projects, and ELIXIR Node activities. This Community has already held a joint meeting to select the key priorities and use cases (see Tables 2 and 3), resulting in this positioning paper. The ELIXIR Toxicology Community will continue to expand and search for contributors with relevant expertise as the community and activities mature. By bootstrapping from Open Science approaches developed in aforementioned projects ( *e.g.* Open PHACTS ^53^), the new Community will focus on mutual benefit, an open and inclusive community, solving practical community problems. The goal is not to design domain-specific approaches, but a pragmatic approach that provides FAIR and open tools from the start, allowing all toxicology and neighbouring communities to benefit from these harmonized solutions. The inclusive community will involve the existing sub-communities in pharmaceuticals, *e.g.* from eTOX, transQST, ^54^ and eTRANSAFE, ^16^ cosmetic ingredients, *e.g.* from SEURAT-1, high and low-volume chemicals, *e.g.* from HBM4EU and soon from Horizon Europe Partnership for the Assessment of Risk from Chemicals (PARC), ^47^ or from different Cefic-LRI (Lang-Range Research Initiative) projects, and nanomaterials, via the NanoSafety Cluster, building on shared needs and community solutions and strongly aligned to other ELIXIR communities. Open licensing and interfaces (ontologies, standards, formats) will encourage new solutions and collaborations, which will be accessible to any organisation and every project within and outside Europe. This will allow close interoperability with toxicology communities outside Europe that also use open approaches, while at the same time allowing compatibility with closed approaches too. This dual model has been demonstrated successfully in recent projects. A prioritized roadmap is essential; the label “ELIXIR Community” would enable us to set priorities at a level above the individual projects. Existing components that will give this Community an initial boost, include, software ( *e.g.* AMBIT with OpenTox API ^55^), databases ( *e.g.* diXa, eNanoMapper, AMBIT-LRI, NORMAN-SLE, MassBank), ontologies ( *e.g.* the eNanoMapper ontology, ^56^ AOP ontology ^57^), interoperability concepts ( *e.g.* annotation of OpenAPIs, in collaboration with bio.tools, semantic structural searches with IDSM), teaching/education material (Bioschemas annotation of outreach activities, in collaboration with TeSS), and virtual infrastructures ( *e.g.* OpenRiskNet Virtual Research Environment and the NORMAN Digital Sample Freezing Platform). However, each of these approaches would benefit from integration in the ELIXIR Platforms (see examples in Table 1) and with Core and National Resources. Various existing ELIXIR Communities need similar solutions, *e.g.* for chemical structure handling, toxicology needs proteomics and metabolomics, toxicology involves human data, and ecotoxicology has significant impact on crops and health.

**Table 2.** Examples of existing and anticipated collaboration.

	Existing collaboration/Reuse	Anticipated collaboration
Tools	Semantic annotation of services ( *e.g.* bio.tools)	Alignment with BioContainers of toxicology workflow efforts ( *e.g.* OpenRiskNet)
Data	diXa was co-developed by the CHEMBL-EBI team, and builds on ArrayExpress an ELIXIR Core Data Resources	Better adoption of these Resources
Compute		Better and more sustainable compute infrastructure reuse of core elements (ELIXIR AAI, modelling toolsets)
Interoperability	FAIR and RDM standards have already been adopted by various projects	Registries of toxicology tools need integration with FAIRsharing; There is a huge identifier mapping service need (also for ontology mapping and chemical (sub)structures)
Training	Bioschemas annotated tutorials	Several projects have training tasks that could be added to TeSS, already partially automated

**Table 3.** The Toxicology Community roadmap is roughly defined by three themes (steps). Each step comes with a 10-year aim, further detailed with possible work that could be done in ELIXIR activities.

Roadmap step	10-year aim	Possible implementation study aim & general criteria
Disseminate existing open solutions (data, database software, models, ontologies, standards, or otherwise) developed among receptive toxicology projects, allowing the advantages of these solutions to become visible to the whole toxicology community	1. Integrating data types across toxicological exposure, biological, Adverse Outcome Pathways, chemical prioritization, hazard assessment, and risk assessment. Data spanning the toxicological domains, properties, exposure, kinetics and dynamics, that support hazard and risk assessment. Approaches include QSAR, QSARDB, ^65^ *in vitro* kinetics, kinetic modelling (toolboxes and parameter estimation), AOPs and Key Event measuring method data results, supporting read-across.	(a) Better adoption of the ELIXIR core and national node resources. (b) Share teaching material about data exchange between resources on TeSS. (c) Disseminate the need of linking database content, *e.g.* with identifier mapping and solutions to match entries between databases
	2. Interoperable Software and Predictive Models	(a) Explore technical connections between existing platforms ( *e.g.* OpenRiskNet) and the Compute Platform and make them more interoperable, working with ELIXIR Interoperability Platform. (b) Wider adoption of the EDAM ontology ^66^ and OpenAPI (c) Explore Common Workflow Language (CWL), port a risk assessment case study as CWL workflow
	3. Ontology and Standards	(a) Adoption of standards like InChI/InChIKey (b) Bioschemas adoption by web databases, and listing in Bioschemas Live Deploys (c) Semantic annotation of assays, cell lines, and Standard Operating Procedures (d) Encourage ontology reuse with dissemination of entering ontology terms as free text with autocorrect, autocomplete and autolookup ( *e.g.* Clinical Data Interchange Standards)
Connect and stimulate cross-project collaborations by making all toxicology research output FAIR.	1. FAIRify existing community resources and solutions for findability and easier reuse	(a) Register all Toxicology resources in FAIRsharing ^11^^,^^10^ and/or the Registry of Research Data Repositories ^67^ to build an awareness and overview of ongoing activities and infrastructures (b) Link up with non-toxicology communities, such as GO FAIR and CDISC (c) Use LabLinks to link Toxicology resources to EuropePMC
	2. Increase FAIRness of relevant data	(a) Register/connect chemicals between PubChem, CompTox, ChEMBL, ChEBI, MolMeDB, IPCheM, IUCLID, HBM4EU, etc and increase integration of toxicologically-relevant information in open resources (b) Develop citation standard for these solutions (c) Integrate kinetics databases and databases needed for kinetic modelling (d) develop and deploy tool boxes that make data registration in repositories easy ( *e.g.* analogous to OneDep in PDB)
	3. Inventorize Question-and-Answer (Q/A) platforms and sub-communities ( *e.g.* tags) on those platforms where people can learn about solutions	(a) Identify how/where toxicologists are asking questions and what their practices are (b) Define tags to be used cross-platform for toxicology and disseminate these among three Q/A platforms
	4. Develop journal editorial standards for minimal reporting standards for describing the chemical entities in deposition databases and compact identifiers in the main text	(a) Reach out to editors. Make an “easy to refer to” guideline available. Find enough journals to join/support this and point to these guidelines. (b) Define a simple format for reporting names and InChI (Keys) and/or SMILES. Consider the possible ontologies for study descriptions for other things often used in toxicology research and toxicity testing (like Salmonella Typhimurium strains, cell systems, LD50 tests) (c) Advocate using compact identifiers (structured as database:dbID, *e.g.* uniprot: P1234) that are easy to use and human readable and that can be resolved by adding links to identifiers.org or n2t.net.
Design and ideally develop missing open community standards to support common interest (Open educational resources, ontologies, APIs, data formats, etc) in advancing toxicology research.	1. Engage with existing (ELIXIR) solutions, like Bioschemas and Bio.tools, ELIXIR AAI, Galaxy including these from connected organisations like RDA, Global Alliance for Genomics and Health (GA4GH), European Open Science Cloud (EOSC), and identify and communicate missing features.	(a) Continue semantic annotation of service APIs ( *e.g.* at bio.tools) and discuss limitations with service and solution providing communities (b) Semantic annotation of training materials, databases, etc with Bioschemas JSON-LD and discuss found limitations (c) ELIXIR AAI rollout in Toxicology platforms (already on OpenRiskNet platform as an example to build from) and report on experiences
	2. Sharing of standard operating procedures (SOPs)	(a) Explore existing solutions to share SOPs ( *e.g.* protocols.io) and discuss limitations
	3. Continued evaluation by adoption of existing ontologies for annotation of toxicology research output	(a) Work with other communities on meta ontologies and mechanisms for (re) creating those from underlying ones and adding dedicated parts. With automated updates and feedback to integrate changes in the base ontologies, *e.g.* Experimental Factor Ontology and the food & nutrition (Ontology of Nutritional Studies ^68^).
	4. Communicate and where possible disseminate FAIR data entry and/or study capture with matching software and/or templates to bench toxicologists	(a) Engage with ELIXIR Training and explore solutions to repurpose existing solutions for data capturing better known in the ELIXIR Toxicology Community ( *e.g.* BioSamples, NanoSafety Cluster templates) (b) Make sure we can capture study designs based on templates can be used for toxicology studies (c) Adopt DataCite practices

The following projects have been adopting and integrating FAIR toxicology concepts, but need integration with ELIXIR Platforms and Communities: eTOX, NanoCommons (NanoSafety Cluster), EU-ToxRisk, OpenRiskNet, OpenTox Foundation, Open PHACTS Foundation, and the diXa platform. Many other projects have a specific scientific focus but also need integration and some will work on the FAIR concepts. A non-exhaustive list is ACEnano, SmartNanoTox, HeCaToS, NewGeneris, EnviroGenoMarkers, EXPOsOMICS, HELIX, ASAT, ^58^ PATROLS, and HEALS, as well as new projects *e.g.* new Horizon 2020 projects RISK-HUNT3R and HARMLESS and the new Horizon Europe project PARC. Companies and organisations will profit from this Community either as users or as providers of services on top of the infrastructure, including ECHA (FI), Edelweiss Connect (CH), IdeaConsult Ltd. (BG), Misvik Biology (FI), TNO (NL), Seven Past Nine (SI), and the Swedish Academic Consortium for Chemical Safety (SwACCS, SE). Industries showed a strong interest in toxicology, demonstrated by their activities: Cosmetics Europe was participant in the SEURAT-1 cluster; chemical industries ( Nanotechnology Industries Association) are participant of the NSC; chemical branch organisation ( Cefic) funds LRI projects around toxicology; pharmaceutical industries funds toxicology research via IMI projects like eTOX, eTRANSAFE, and Open PHACTS. The Research Data Alliance (RDA) organized a workshop recently about integration of toxicogenomics resources, ^59^ and collaboration with international organizations has already been established with, for example, the CompTox Chemicals Dashboard team of the US EPA ^60^ and PubChem from the US National Institutes of Health. ^61^

## Competing interests

None.

## Grant information

This work received funding from the European Union’s Horizon 2020 research infrastrcuture programme via the OpenRiskNet project under grant agreement No. 731075. This project has received funding from the European Union’s Horizon 2020 research and innovation programme under grant agreement No. 681002 (EUToxRisk). This project has received funding from the European Union’s Horizon 2020 research and innovation programme under grant agreement No. 814572. SN acknowledges BMBF funding under grant number 031L0107. This work was supported by OBERON ( https://oberon-4eu.com), a project funded by the European Union’s Horizon 2020 research and innovation program under the grant agreement No. 825712. This work was supported by the European Union,’s Horizon 2020 research and innovation program HBM4EU, grant agreement No. 733032 ( https://www.hbm4eu.eu). Supported by the European Union’s Horizon 2020 programme under the Marie Skłodowska-Curie grant agreement No. 859891. Where authors are identified as personnel of the International Agency for Research on Cancer/World Health Organization, the authors alone are responsible for the views expressed in this article, and they do not necessarily represent the decisions, policy, or views of the International Agency for Research on Cancer/World Health Organization. This work was supported by the European Union,’s Horizon 2020 research and innovation program HARMLESS, grant agreement No. 953183. This work was supported by the European Union,’s Horizon 2020 research and innovation program Gov4Nano, grant agreement No. 814401. This work was supported by the Swedish Fund for Research Without Animal Experiments under grant number N2020-0005. This work was supported by the European Union,’s Horizon 2020 research and innovation program NTS-EXPOSURE, Grant agreement ID: 896141. This work was supported by the European Union,’s LIFE program LIFE-APEX, Grant agreement ID: LIFE17 ENV/SK/000355. This study was funded by the German Environment Agency within the PHION project (grant number 3718 674150). The Data Readiness Group is supported, in this ELIXIR Community, by the H2020 Precision Toxicology project (H2020-EU 965406). The Data Readiness Group is supported, in this ELIXIR Community, by the Wellcome ISA-InterMine project (208381/A/17/Z). The Data Readiness Group is supported, in this ELIXIR Community, by the Wellcome FAIRsharing project (212930/Z/18/Z). This work received funding from the European Union’s Horizon 2020 research and innovation programme RiskGONE Project under grant agreement No. 814425. NR’s research is funded by a Miguel Servet contract (CO19/00060) from Instituto de Salud Carlos III, cofinanced by the European Union. UM (Uni. of Tartu) is grateful for support to Ministry of Education and Research, Republic of Estonia through Estonian Research Council (grant number IUT34-14) and to European Union European Regional Development Fund through Foundation Archimedes (grant number TK143, Centre of Excellence in Molecular Cell Engineering). Development and Implementation of a Sustainable Modelling Platform for NanoInformatics. Linking LRI Ambit chemoinformatic system with the IUCLID substance database to support read-across of substance endpoint data and category formation. This work was supported by the French Ministry of Research and National Research Agency as part of the French MetaboHUB, the national metabolomics and fluxomics infrastructure (Grant ANR-INBS-0010). This project has received funding from the European Union’s Horizon 2020 research and innovation program under grant agreement GOLIATH No. 825489.

## Disclaimer

• Where authors are identified as personnel of the International Agency for Research on Cancer/World Health Organization, the authors alone are responsible for the views expressed in this article, and they do not necessarily represent the decisions, policy, or views of the International Agency for Research on Cancer/World Health Organization.
• The views expressed in this manuscript are solely those of the authors and do not represent the policies of the U.S. Environmental Protection Agency. Mention of trade names of commercial products should not be interpreted as an endorsement by the U.S. Environmental Protection Agency.
