# Supplementary material for: ELIXIR and Toxicology: a community in development
Source: F1000Res. 2023 Oct 3;10:ELIXIR-1129. Originally published 2021 Nov 8. [Version 2] doi: 10.12688/f1000research.74502.2 (PMC10568213; doi:10.12688/f1000research.74502.2)
Supplement: Peer review history

# Reviewer report: Reviewer response for version 2

**DOI:** 10.5256/f1000research.156142.r211615

**Published:** 2023-10-11

**Authors:** Christopher Southan

**Affiliations:** 1 Data Sciences,, Medicines Discovery Catapult, Macclesfield, UK; 2 Deanery of Biomedical Sciences, University of Edinburgh, Edinburgh, UK

The authors have addressed my comments in this revision I thus hereby approve it.

Is the topic of the opinion article discussed accurately in the context of the current literature?

Yes

Are arguments sufficiently supported by evidence from the published literature?

Yes

Are all factual statements correct and adequately supported by citations?

Yes

Are the conclusions drawn balanced and justified on the basis of the presented arguments?

Yes

Reviewer Expertise:

Bioinformatics and cheminformatics (see LinkedIN)

I confirm that I have read this submission and believe that I have an appropriate level of expertise to confirm that it is of an acceptable scientific standard.

# Reviewer report: Reviewer response for version 1

**DOI:** 10.5256/f1000research.78265.r124031

**Published:** 2022-04-08

**Authors:** Marta Eide

**Affiliations:** 1 Centre for Digital Life Norway, Bergen, Norway; 2 Department of Biological Sciences, University of Bergen, Bergen, Norway

In this opinion article, the authors describe the establishment of a new ELIXIR Toxicology Community in order to enable the transition from traditional disease-specific observational models to target-specific predictive models. The article introduces the rapidly developing field of toxicology and the interoperability issues arising in combining chemical and biochemical analysis, biological data, and kinetic modelling. The authors argue for “ *clear, standardized guidelines for data capture, approaches to integrate and connect across multiple datasets, clear data licensing for these data repositories, and tools that support accessing the data*”. The ELIXIR Toxicology Community is suggested to 1) connect the different user communities (including industry, governmental agencies, NGOs, and more), 2) strongly contribute to link between, and harmonize the already existing relevant infrastructure projects, resources, and activities, and 3) provide useful tools, guidelines, and training.

 Acknowledging the vast number of chemicals that are currently being produced, and the limited knowledge of the consequences of exposure to humans and the environment, this is a timely and important effort. From my impression, an ELIXIR Community is a good tool to drive and coordinate such an international initiative.

 I find the article comprehensive and well-written, and their arguments well discussed. However, I have some points that I hope the authors will take into consideration when taking the initiative further:

1. It is early stated that “ *in this ELIXIR Community, the focus will be primarily on the protection of human health*”. I would argue that this is an unfavorable restriction for three reasons:
  - It disregards the ‘OneHealth approach’ recognized by WHO, FAO, OIE, and UNEP, which is defined as “ *an integrated, unifying approach that aims to sustainably balance and optimize the health of people, animals and ecosystem. It recognizes the health of humans, domestic and wild animals, plants, and the wider environment (including ecosystems) are closely linked and inter-dependent*” ( One Health High-Level Expert Panel Annual Report 2021). EFSA, ECHA, EMA *et al.* are also organizing a conference on the topic in June 2022 ( https://www.one2022.eu). Seeing that animals and ecosystems are closely linked to human health, for example through foods, their responses to and effects from toxicant exposures are also highly important to study and include in risk assessments.
  - Live animals, as well as animal tissue, cells, and molecules, are often used as models for toxicological studies. As the toxicological defense systems are highly preserved through evolution, they can give a good indication of human outcomes. Thus, it is important to also ensure supporting infrastructure for integrating these as well.
  - Ontologies, standards, and data resources (like omics repositories, data analysis tools, etc.) are often already overlapping.
2. Although I welcome different stakeholders being identified as “user communities”, I am concerned by the, from my understanding, lack of industry and governmental/administrative agencies in the author list of this article. I believe that if the aim of improving FAIR data integration for safety to enable ultimate risk assessments is to be met, it is eminent to include non-academic perspectives, experiences, and concerns into the initiative from early on. Experiences from the PARC project could for example be useful in how to approach this.
3. From my experience, the field of toxicology in general has limited experience with FAIR data management and is not familiar with the resources that are in place. I see that toxicologists’ communication, dissemination, and training are mentioned in the proposed roadmap, but I am missing a more targeted approach to engaging with the communities. For example, such activities could include organizing toxicology-specific workshops, presenting at toxicology conferences, and targeted information through different channels. As the proposed ELIXIR Community seems to have a good overview of ongoing initiatives and infrastructures and a good anchoring in the scientific community, a mapping of their target audience and users in the field could be suggested as a starting point.  
4. As mentioned in the paper, systems biology approaches are also getting implemented in toxicology, i.e. systems toxicology (described in Sturla *et al.,* 2014 ^1^. From my point of view, I am missing mentioning of tools for this, such as the FAIRDOM SEEK platform that integrates ISA-structured metadata and workflows with JWS-based biological systems modelling. However, there might already be other solutions for this that I don’t recognize in the paper

Is the topic of the opinion article discussed accurately in the context of the current literature?

Yes

Are arguments sufficiently supported by evidence from the published literature?

Yes

Are all factual statements correct and adequately supported by citations?

Yes

Are the conclusions drawn balanced and justified on the basis of the presented arguments?

Yes

Reviewer Expertise:

Environmental toxicology, endocrine disruption, bioinformatics, systems toxicology, marine toxicology

I confirm that I have read this submission and believe that I have an appropriate level of expertise to confirm that it is of an acceptable scientific standard.

## References

[1] : Systems toxicology: from basic research to risk assessment. *Chem Res Toxicol* .2014;27(3) : 10.1021/tx400410s 314-29 10.1021/tx400410s 24446777PMC3964730

# Reviewer report: Reviewer response for version 1

**DOI:** 10.5256/f1000research.78265.r124032

**Published:** 2022-03-21

**Authors:** Christopher Southan

**Affiliations:** 1 Data Sciences,, Medicines Discovery Catapult, Macclesfield, UK; 2 Deanery of Biomedical Sciences, University of Edinburgh, Edinburgh, UK

This wide-ranging review circumscribes an important data integration task with a major cat-herding dimension. Whilst also appearing herculean, this esteemed author collective fully understands what they are letting themselves in for and I wish them the best for this endeavor.  It is, of course, early days, but I will make a few points (whether these might be addressed in a revision and blending in what other reviewers may come up with I will leave to the authors)

  

1. Harvesting tox data from the extant and future literature seems to neither be specifically addressed nor proposed via direct interactions with the pharmaceutical companies generating most of it. Standardised data from their large historical internal sets only surfaces sparsely and heterogeneously public databases. Companies such as LahsaVtic, Instem, and ToxPlanet claim to have large databases compiled from the literature. Might ChEMBL come into the frame here if they could strategically increase their toxicology data extraction from the literature, both prospectively and retrospectively? (So far with only 24 assays)
2. My impression is there are simply too many resources mentioned for realistic overarching harmonisation. Could these be cut back to a smaller “active membership” prioritised by the amount of data they have and will continue to generate?
3. Quote “standardized guidelines for data capture, approaches to integrate and connect across multiple databases”. As we know, this is going to be the really tough bit. It needs the major sources to share not only their data schema and metadata indexing but push for a level of convergence. It has to be said that ELIXIR does not have a good record on this.  Unless I have missed it, none of the core data resources has made any significant changes in their individual (and often decades-old) data models to really enhance interoperability.   
4. PubChem has 113,110 compounds from ~ 30 annotation sources (although most relatively small) indexed under “PubChem Compound TOC: Toxicological Information” and toxicology-related submitting sources cover over 0.5 million substances. In addition, the term “toxicology” matches 3,774 BioAssays covering 86,068 compounds.  This makes PubChem a *de facto* toxicology integration hub. The ELIXIR effort should consolidate and expand this, including where an increase in toxicology curation by ChEMBL could feed into BioAssay and thus become interoperative with the 294,000 biological activity data points already in there.

Is the topic of the opinion article discussed accurately in the context of the current literature?

Yes

Are arguments sufficiently supported by evidence from the published literature?

Yes

Are all factual statements correct and adequately supported by citations?

Yes

Are the conclusions drawn balanced and justified on the basis of the presented arguments?

Yes

Reviewer Expertise:

Bioinformatics and cheminformatics (see LinkedIN)

I confirm that I have read this submission and believe that I have an appropriate level of expertise to confirm that it is of an acceptable scientific standard, however I have significant reservations, as outlined above.
